# Epithelial Cells of Deep Infiltrating Endometriosis Harbor Mutations in Cancer Driver Genes

**DOI:** 10.3390/cells10040749

**Published:** 2021-03-29

**Authors:** Agnieszka Koppolu, Radosław B. Maksym, Wiktor Paskal, Marcin Machnicki, Beata Rak, Monika Pępek, Filip Garbicz, Kacper Pełka, Zofia Kuśmierczyk, Joanna Jacko, Małgorzata Rydzanicz, Magdalena Banach-Orłowska, Tomasz Stokłosa, Rafał Płoski, Jacek Malejczyk, Paweł K. Włodarski

**Affiliations:** 1 Department of Medical Genetics, Medical University of Warsaw, 02-106 Warsaw, Poland; agnieszka.jacoszek@wum.edu.pl (A.K.); mrydzanicz@wum.edu.pl (M.R.); rafal.ploski@wum.edu.pl (R.P.); 2 Postgraduate School of Molecular Medicine, Medical University of Warsaw, 02-091 Warsaw, Poland; rak.beatka@gmail.com (B.R.); monika.pepek@wum.edu.pl (M.P.); filip.garbicz@gmail.com (F.G.); 3 Department of Reproductive Health, Centre of Postgraduate Medical Education, 01-004 Warsaw, Poland; 4 Centre for Preclinical Research, Department of Methodology, Medical University of Warsaw, 02-091 Warsaw, Poland; wiktor.paskal@wum.edu.pl (W.P.); kacper.pelka@wum.edu.pl (K.P.); zofiadomosud@gmail.com (Z.K.); pielasj@gmail.com (J.J.); 5 Department of Tumor Biology and Genetics, Medical University of Warsaw, 02-106 Warsaw, Poland; marmach.marmach@gmail.com (M.M.); tomasz.stoklosa@wum.edu.pl (T.S.); 6 Laboratory of Centre for Preclinical Research, Department of Histology and Embryology, Medical University of Warsaw, 02-091 Warsaw, Poland; mbanach@iimcb.gov.pl (M.B.-O.); jacek.malejczyk@wum.edu.pl (J.M.); 7 Department of Experimental Hematology, Institute of Hematology and Transfusion Medicine, 02-781 Warsaw, Poland; 8 Laboratory for Experimental Immunology, Military Institute of Hygiene and Epidemiology, 01-163 Warsaw, Poland

**Keywords:** endometriosis, laser-capture microdissection, somatic variants, NGS sequencing, endometrial glands, deep endometriosis

## Abstract

Endometriosis is an inflammatory condition manifested by the presence of endometrial-like tissue outside of the uterine cavity. The most common clinical presentations of endometriosis are dysmenorrhea, infertility, and severe pelvic pain. Few hypotheses attempt to explain the pathogenesis of endometriosis; however, none of the theories have been fully confirmed or considered universal. We examined somatic mutations in eutopic endometrium samples, deep endometriotic nodules and peripheral blood from 13 women with deep endometriosis of the rectovaginal space. Somatic variants were identified in laser microdissected samples using next-generation sequencing. A custom panel of 1296 cancer-related genes was employed, and selected genes representing cancer drivers and non-drivers for endometrial and ovarian cancer were thoroughly investigated. All 59 detected somatic variants were of low mutated allele frequency (<10%). In deep ectopic lesions, detected variants were significantly more often located in cancer driver genes, whereas in eutopic endometrium, there was no such distribution. Our results converge with other reports, where cancer-related mutations were found in endometriosis without cancer, particularly recurrent *KRAS* mutations. Genetic alterations located in ectopic endometriotic nodules could contribute to their formation; nevertheless, to better understand the pathogenesis of this disease, more research in this area must be performed.

## 1. Introduction

Endometriosis is an inflammatory condition manifested by the presence of endometrial-like tissue outside of the uterine cavity. Most commonly, the lesions are located in the pelvis and peritoneal cavity; nevertheless, they may be found in distant parts of the body–including the lungs and brain. The most common clinical presentations of endometriosis are dysmenorrhea, infertility, and severe pelvic pain, which significantly lower the quality of life of the affected women. It has been estimated that around 10% of women of reproductive age and up to 50% of infertile women suffer from endometriosis. Because of severe pain during menstruation, hormonal therapy and surgical intervention are often required [1,2,3,4,5,6,7,8].

The classification of endometriosis remains complex as it can be performed based on localization, histology, clinical symptoms; however, these classifications do not predict disease burden, recurrence or prognosis. We confined the basic general classification of endometriosis into three types: superficial peritoneal endometriosis, ovarian endometriomas, and deep endometriosis. Although less commonly recognized than the others, the latter one is the most aggressive type of the disease that is usually localized in the uterosacral ligaments, the rectovaginal space, the upper third of the posterior vaginal wall, the urinary tract, and the bowel [9].

Few hypotheses attempt to explain the pathogenesis of endometriosis; however, none of them have been universally accepted due to insufficient evidence. One of the most widely accepted explanations is Sampson’s retrograde menstruation theory. According to his concept, the endometrial tissue cells translocate to the pelvic cavity during menstruation with the backward flux of menstrual debris through the Fallopian tubes [10]. This hypothesis, however, does not explain all the cases of endometriosis, and although retrograde menstruation occurs in many women, not all of them are affected by endometriosis. Once the ectopic endometriotic lesions are present, multiple mechanisms involved with extracellular matrix remodeling are activated, resembling cancer-like progression and invasive growth [11,12].

There is a growing body of evidence that genetic factors predispose to the development of endometriosis. Genome-wide association studies (GWASs) and genome-wide linkage studies (GWLSs) reported over 10 loci and specific pathways associated with the onset and inheritance of this disease [13,14,15,16]. There have been no reports on individual genes or gene variants unambiguously involved in the pathogenesis of endometriosis. Endometriosis is a disease with diverse phenotypes (i.e., the location of ectopic endometrial tissue), and up-to-date findings indicate that the mechanisms driving its onset can be different in each case, often combining a few hypotheses. Environmental and immune factors further complicate investigations of the pathogenesis of endometriosis.

Attempts to identify somatic mutations that lead to the development of endometriotic lesions proved that some cancer-driving genes are more frequently mutated in ovarian endometriosis [17,18]. Mutations in these genes: *ARID1A*, *PIK3CA*, *KRAS,* are also found in ovarian cancer that is associated with endometriosis [19]. However, the same genes have been found to be mutated in eutopic endometrium in women without endometriosis, leaving the question about their pathogenicity still unanswered.

Deep endometriosis rarely leads to cancer development, and little is known about the role of mutations in the pathogenesis of this type of disease. Therefore, this study aimed to identify somatic mutations in endometrial cells of the deep ectopic lesions and in paired eutopic endometrium to determine if particular gene mutations are associated with the pathogenesis of this disorder.

## 2. Materials and Methods

### 2.1. Patients

Eutopic endometrial tissue, ectopic lesions, and blood samples were collected from 21 women (mean age 33.3 years, SD = 5.3 years, range 25–42 years) with deep endometriosis of the rectovaginal space confirmed by laparoscopic and histopathological evaluation. Patients underwent surgery at the Endometriosis Center of Gynecological Department of St. Sophia Hospital, Warsaw, Poland. All patients gave informed consent to the study, and the investigations were approved and conducted according to the strict guidance of the local Ethics Committees in the Medical University of Warsaw and Military Institute of Medicine.

All patients underwent laparoscopic surgery (Aeskulap 3D EinsteinVision^®^ 2.0, B. Braun, Melsungen, Germany) due to symptomatic deep endometriosis infiltration. Nodules were diagnosed before the operation by bimanual gynecological examination and by transvaginal ultrasonography with endovaginal gel using the technique in accordance with IDEA rules [20].

All cases in this study were classified during surgery as stage III/IV of endometriosis according to the revised criteria of the American Society Reproductive Medicine (rASRM) [2] and B1/B2 in the revised Enzian classification [9]. The study was performed on specimens taken solely from deep lesions of uterosacral ligaments (deep endometriosis). Surgery was performed during continuous progestin therapy or on days 5–10 of the menstrual cycle. None of the patients suffered from any other inherited or chronic disorder, hematologic malignancy, or cancer. All the patients had never been pregnant before surgery, but two of them conceived and gave birth after surgery (EEP001, EEP0017).

### 2.2. Collection and Preparation of Endometrium, Endometrial Tissue Specimens, and Peripheral Blood

Samples of endometrial deep-infiltrating lesions were excised by the bipolar cut (LAP BiSect, Erbe, Tübingen, Germany) during laparoscopy from uterosacral ligaments. Samples of eutopic endometrium were collected during surgery by trans-cervical endometrial aspiration biopsy (Pipelle, Cornier). Samples of tissue used for bulk tissue sequencing were preserves in RNA-later reagent (Sigma-Aldrich, St. Louis, MO, USA). Tissue samples prepared for laser-capture microdissection were rinsed in saline, immediately immersed in OCT medium (Optimal cutting temperature compound, Tissue-Tek, Torrance, CA, USA), and snap-frozen. Peripheral blood was collected to EDTA vials (BD Vacutainer, Franklin Lakes, NJ, USA) and immediately frozen. Tissue and blood samples were stored at −80 °C until further processing. Each patient provided a set of tree samples originating from blood, endometrium and deep nodule.

### 2.3. Bulk Tissue Samples Preparation

Endometrial bulk tissue samples from 8 individuals were rinsed in PBS and homogenized using Omni tissue homogenizer (Omni International, Kennesaw Georgia, Georgia). Genomic DNA was then extracted using the salting-out procedure as was described before [21]. Libraries were prepared for sequencing using TruSeq Exome Kit (Illumina, San Diego, CA, USA) according to the manufacturer’s protocol.

### 2.4. Tissue Preparation and Laser-Capture Microdissection (LCM)

**Figure 1:**
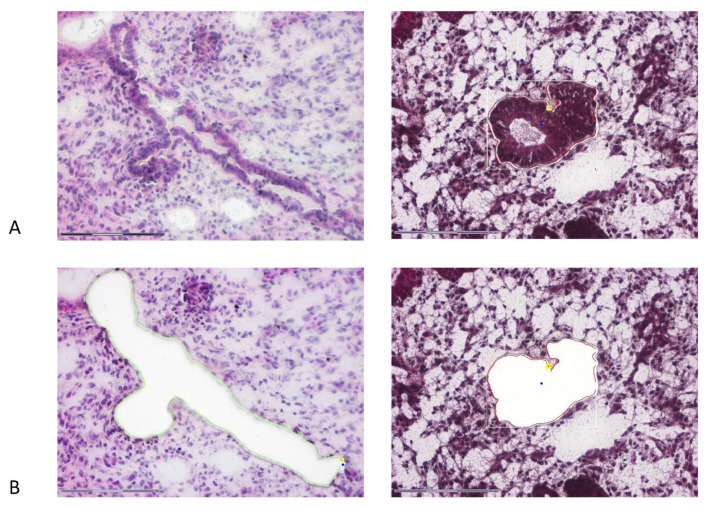
A single representative endometrial gland before (**A**) and after (**B**) laser microdissection from 8 µm tissue slice. For a demonstration of gland mapping, eosin and hematoxylin staining was used on this sample (on the left). For DNA extraction, sections were stained with hematoxylin only (on the right).

Paired tissues specimens of 13 patients (eutopic endometrial biopsies and ectopic nodules) were cut with a cryostat (CM 1860, Leica, San Francisco, CA, USA) into 8 µm sections, which were mounted on glass slides (SuperFrost^®^, Menzel, Berlin, Germany) and underwent standard H&E staining, followed by an initial assessment of the presence of endometrial epithelium in both samples. After confirmation, the samples were once again cut with a cryostat. At least 9 sections (8 µm thick, 3 per slide) were heat-mounted on PEN membrane-coated glass slides (MembraneSlide 1.0, Zeiss PALM^®^, Bernried, Germany). Prior to mounting, the membranes were incubated for 30 min under UV light to improve adhesion. The mounted sections were stained with a brief, modified Hematoxylin staining protocol: incubated for 1 min in −20 °C ethanol, dried for 30 s at room temperature, washed with RNAse/DNAse-free water, incubated for 30 s in Meyer Hematoxylin, washed again with RNAse/DNAse-free water, rinsed until bluing in Scotts Tap water, washed again with RNAse/DNAse-free water and 100% ethanol twice, dried for 3 min until further steps were undertaken. Eosin was omitted due to its possible inhibiting effect on downstream reactions. Then, laser-capture microdissection (LCM, Zeiss PALM MicroBeam, Bernried, Germany) was performed. An example of a representative slide before and after the LCM is shown in Figure 1. The cutting energy was optimized and set to 46–55 units and catapulting energy set to 67 units. Only LED illumination was used to decrease samples’ overheating and degradation. 3 slides containing 9 sections were scanned with a 5× magnification. 3 glands from each section were marked and chosen for microdissection with both cutting and centered laser pressure catapulting program under 40× magnification. Catapulted glands were collected with opaque 500 µL AdhesiveCaps (Zeiss, Oberkochen, Germany) and stored at −20 °C until further processing. Each tube contained 3 endometrial glands. From each sample, glands were collected in triplicates to 3 independent tubes.

### 2.5. DNA Extraction

Peripheral blood DNA was isolated using a QIAamp DNA mini kit (QIAGEN) according to the manufacturer’s protocol. DNA from laser microdissected tissues was extracted and simultaneously amplified using RepliG mini whole-genome amplification (WGA) kit (QIAGEN). WGA was performed simultaneously on three samples (each one containing 3 glands). Tissue samples were qualified for further experiments when the total amount of extracted and amplified DNA was above 1000 ng. One of three LCM samples with the highest amount of DNA was qualified for further sequencing. Accordingly, for each patient, a final 3 samples were obtained, each representing one of the distinct tissue compartments.

### 2.6. Somatic Variants Detection–Genomic DNA Library Preparation and Sequencing

The mutational background was determined using targeted sequencing of approximately 10Mb of exonic regions of almost 1300 cancer-associated genes (Supplementary Table S1). Genomic DNA libraries were prepared using a KAPA HTP library preparation kit, multiplexed before solution-based custom capture (Roche NimbleGen, Pleasanton, CA, USA). All libraries were assessed using Bioanalyzer and Qubit and paired-end sequenced (2 × 100 bp) on Illumina HiSeq 1500 to obtain the average mean coverage of 150×.

### 2.7. Data Analysis–Bulk Tissue and LCM Tissue

Raw NGS data were processed as previously described [22] with Hg19 genomic build used for alignments. In order to detect somatic mutations in bulk tissues, MuTect and VarScan2 were used. In LCM tissues, somatic mutations were detected by manual comparison of the processed data–ectopic or eutopic sample against the blood sample as a reference. Somatic variants in endometrial tissues were identified based on their absence in the reference peripheral blood sample. Selected variants were verified manually using an integrated genomics viewer (IGV). For further analyses, only non-silent variants were included. A variant was considered as present when at least two reads with variants were reported in a sample. A variant was considered absent in a sample when the coverage in a particular location was >20×, and no change of nucleotides was observed in this location. Variants detected in all laser microdissected samples in each patient were presented in Table 1.

Cancer driver genes were listed based on the mutational cancer drivers database (IntOGen). Genes harboring somatic variants were divided into driver and non-driver gene groups according to the consolidated endometrial and ovarian cancer driver gene lists.

### 2.8. Variant Verification–NGS Library Preparation and Sequencing

Selected variants were verified by the amplicon deep sequencing (ADS) method. Specific primers were designed for each variant selected for verification and used to obtain >300 bp PCR products containing the region of interest. NGS libraries were prepared using Nextera XT (Illumina, San Diego, CA, USA) and paired-end sequenced on Illumina HiSeq 1500 to obtain coverage of >2000×. The verification procedure was performed on all the available samples from each patient–blood DNA sample, LCM samples that underwent NGS sequencing, as well as the remaining samples with successfully extracted DNA from LCM ectopic or eutopic tissue, which were not included in NGS sequencing.

### 2.9. Statistical Analysis

Statistical analysis was performed using GraphPad Prism version 8.0.0 for Windows (GraphPad Software, San Diego, CA, USA). Descriptive statistics, Chi^2^, and Fisher’s exact tests were used when appropriate. A *p*-value < 0.05 was considered statistically significant.

## 3. Results

### 3.1. Detection of Somatic Variants in Bulk Tissue Samples

Sequencing of DNA extracted from bulk tissues resulted in a mean coverage of 46×. It did not reveal any somatic variants in comparison to peripheral blood. Therefore, we decided to analyze sequences obtained selectively from epithelial cells microdissected from tissues.

### 3.2. Detection of Somatic Variants in LCM Tissue Samples

Custom panel NGS sequencing was performed on samples obtained from 13 patients–eutopic and ectopic glandular epithelium (GE) as well as peripheral blood as a reference. Sequencing resulted in the average mean coverage of 159×, and the average percentage of the target covered at least 20× was 82%. Fifty-nine non-silent variants were detected in all the samples, including 24 variants in ectopic GE, 31 in eutopic GE, and 4 in both GE.

All detected somatic variants were of low mutated allele frequency (<10%) and are presented in Supplementary Table S1. In one patient (EEP003), we did not detect any somatic variants. Two other patients (EEP001, EEP002) did not harbor any somatic variants in the eutopic tissue. Only four patients harbored identical somatic variants in both eutopic as well as ectopic endometrial tissue: *HERC2* in EEP001, *JAK2* in EEP005, *DYSF,* and *TROAP* in EEP015 (Supplementary Figure S1). ADS verification excluded three of them (*HERC2*, *JAK2*, *TROAP*) as they were absent in all the samples but confirmed the presence of the variant in one out of two eutopic samples (Supplementary Figure S2). Genes mutated in more than one patient were *ATRX*, *RYR1*, *DNAH7,* but no recurrent variants were observed. One ectopic GE sample (EEP009) carried a p.Glu271Lys/c.811G > A *TP53* variant. One *KRAS* p.Gly12Asp/c.35G > A variant was detected in an ectopic GE sample (EEP002) (Supplementary Figure S3). ADS verification confirmed the presence of the *TP53* variant in both available WGA ectopic samples and the *KRAS* variant in one of two ectopic samples (Supplementary Figure S2).

**Figure 2:**
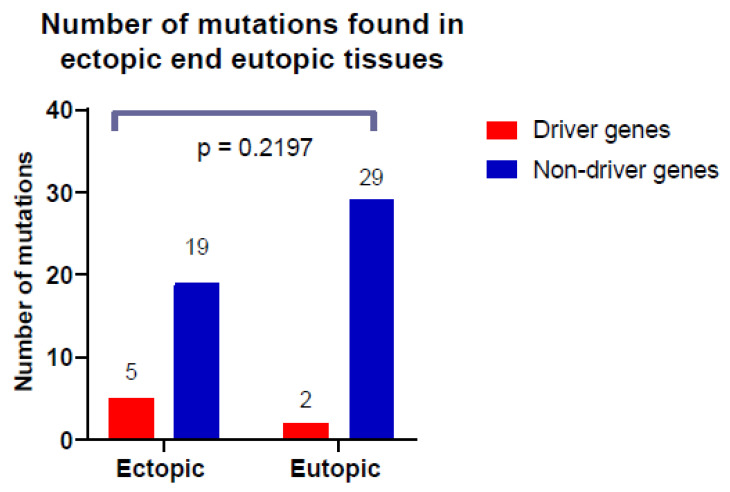
Number of cancer driver and non-driver mutations found in ectopic end eutopic tissues. Calculated using Fisher’s exact test.

Out of 1296 genes and gene hot spots represented in our panel, 85 were classified as driver genes, and the remaining 1211 genes were classified as non-driver genes. Overall, in the ectopic tissue, out of 24 detected variants, 5 were located in driver genes, while the remaining 19 were in non-driver genes. Therefore, the chance of driver gene and non-driver gene mutation in the ectopic tissue was 0.059 (5/85) and 0.016 (19/1211), respectively. The difference in the frequency of mutation occurrence among driver and non-driver genes was statistically significant for the ectopic tissue (*p* = 0.0199*), as is shown in Figure 2).

In the eutopic tissue, out of 31 detected mutations, 2 were located in driver genes and 29 in non-driver genes. Therefore, the chance of mutation in both types of genes was equal (2/85 ≅ 29/1211 ≅ 0.024). The differences in the frequency of occurrence of the driver genes mutations and non-driver genes mutations in eutopic tissue were statistically insignificant (Figure 2).

We also compared the occurrence of driver and non-driver genes mutation between ectopic and eutopic tissue, but it did not reach statistical significance (*p* = 0.2197), which is shown in Figure 2.

## 4. Discussion

The formation of ectopic lesions in endometriosis has been investigated extensively, leaving no convincing evidence for a single, universal mechanism responsible for the development of this disease. On the contrary, data accumulated in recent decades point to multiple dysregulated molecular pathways found in cells of endometriotic foci (reviewed in [23]). Local tissue remodeling, proinflammatory cytokines release, and immune cell involvement are observed in endometriotic tissue, along with enhanced proliferation and survival due to apoptosis inhibition. The mechanisms of these aberrations are common with those that are governing malignant transformation. Cell–cell and cell–matrix adhesion molecules are engaged both in cancer tumors and in endometriosis [12]. Similarly, epigenetic modifications of genes in endometriosis are also found in cancer [24]. Basing on these analogies, several groups aimed at identifying genes that are associated with the occurrence of endometriosis.

In accordance with previous reports [13,14,15,16,17,25], our study shows that there is no single gene or mutation responsible for endometriosis development. We report that individual glands in endometriotic tissues carry different mutations aiming at different molecular backgrounds originating from endometrial tissue. The use of laser microdissection allowed obtaining samples composed of epithelial cells of one endometrial gland, which possibly arises from a single stem cell located in its niche at the basal layer. Upon sequencing of such genetically homogenous samples, we have identified numerous somatic mutations in deep infiltrating ectopic lesions of endometriosis. Since mutations are found only in epithelial glands but not in the endometrial stroma [26], bulk tissue sequencing is clouded by this component. Thus, our initial approach to detect somatic variants in endometrial tissues by sequencing bulk tissue samples had to be replaced with LCM for more specific results obtained selectively from the endometrial epithelium. The verification study showed that despite the high depth of sequencing and sophisticated bioinformatical analysis, several variants revealed by good quality NGS reads turned out to be artifacts. This could be due to the lack of WGA fidelity, PCR artifacts occurring during NGS library preparation, or differences between tissue sections. This could explain why the *KRAS* variant was confirmed only in one out of two available DNA samples of the same tissue. An alternative explanation was recently offered by Yachida et al., who demonstrated uneven spatial intratumor heterogeneity of KRAS mutant allele expression within one endometriotic sample [27]. Nevertheless, the *TP53* variant was confirmed in both available WGA ectopic samples.

Even though all the patients recruited to our study had no history and no features of neoplastic disease, the results revealed mutations in known cancer driver genes, especially in ectopic lesions. These mutated genes included *KRAS*, *TP53*, and *ATRX*. Independent studies confirmed mutations in cancer driver genes in deep infiltrating lesions. Our results converge with other reports, where cancer-related mutations were found in endometriosis without cancer, in particular recurrent *KRAS* mutations [17,25,26]. In our patients, we found only one, single p.Gly12Asp *KRAS* variant that was mentioned in both previously published reports. This variant affects a frequently mutated hot spot–amino acid 12. Nevertheless, it was not recurrent, and we have not found any other variant in genes mentioned in these papers (*ARID1A PIK3CA*, *PPP2R1A*). Thus, our results contribute to consider *KRAS* variants, in particular those located in the 12th amino acid, as linked to endometriosis.

Although non-ovarian deep infiltrating endometriosis rarely undergoes cancer transformation (transformation risk is estimated to be less than 1%) [26,28], variants in genes strongly linked to cancer (*KRAS* in EEP003 and *TP53* in EEP009) could also foreshadow the neoplastic transformation in these patients’ ectopic lesions. Our data show that in ectopic GE, mutations in driver genes occur significantly more often than in non-driver genes. This phenomenon was not observed in eutopic tissue, where the variants in driver and non-driver genes occurred with almost the same frequency. Although, to date, there is no direct link between endometriosis and endometrial or ovarian cancer, variants in these genes are commonly found in other diseases, including endometrial cancer or ovarian cancer. However, this fact needs to be supported by more clinical evidence, including the follow-up of the women with endometriosis.

Cheng W et al. for the first time, provided a model to test clinically validated driver genes in a mouse model predisposed to endometriotic lesion formation by the activation of mutated *KRAS* in donor endometrial epithelium and stroma [29]. However, there is still an open question of whether a single mutation is sufficient for cancerous or endometrioid transformation. Further research, both in vitro and on an animal model, is essential to identify and explain the role of *KRAS* mutations in endometriosis. Considering that isolated driver mutations of ARID1A or PIK3CA transduced into normal endometrial epithelium have no gross phenotypes, but in combination, the atypical phenotype arises [30], a similar requirement may apply to expose the phenotype of *KRAS* mutation in the endometrial epithelium. Moreover, in normal endometrium and in ovarian endometrioma, similar driver mutations are found; however, the mutant allele frequency is higher in the latter [17,31].

Wu Y et al. described over 900 genes, which were deferentially expressed in ectopic and eutopic endometrioid tissues. They suggested the importance of over seventy pathways with over one hundred genes involved in the disease pathogenesis [32]. Recent data, based on proteomic analysis of ectopic and eutopic tissues, showed that ectopic endometrial stromal cells exhibit reduced apoptotic potential, altered immune function, as well as increased cellular invasiveness and adhesiveness, which are known as cancer features [33]. What is more, gene expression analysis revealed numerous dysregulated genes commonly involved in oncogenesis, including regulators of cell cycle, mTOR, MAP, TGF-β, WNT, or JAK/STAT pathways [32].

Although to date, endometriosis is considered to be a nonmalignant inflammatory disease [34], Anglesio et al. hypothesize that deep infiltrating endometriosis could be considered as a metastatic disease; this is supported by cases of endometrial tissue in extremely rare locations like the brain or lungs [26]. Their research discovered the same mutations within endometriotic tissue in three different distant places. Some of the mutations reported in our study were present both in ectopic and eutopic lesions like *JAK2* or *TROAP*. Although these variants have not been confirmed in the verification study, more research is needed to investigate whether variants shared by both eutopic and ectopic endometrial tissue exist and could indicate the mutual origin of those tissues. Thus, an extended investigation that would analyze more samples from differently located lesions in each patient should be carried out to support this hypothesis.

An interesting hypothesis, a so-called stem-cell-related theory, provides an understandable explanation for single-cell originating endometrial metastases in distant localizations. Epithelial cells in a retrograde menstrual flux present different developmental stages that indicate potential stem-cell-like properties. Therefore, these cells could contribute to the formation of lesions distinct from the uterine cavity cells [35]. This theory could provide a satisfactory explanation of the same mutations carried by eutopic and ectopic endometrioid tissues in patients. In our study, we did not find variants shared by both tissues of the same patient. Therefore, our results cannot support this hypothesis. Nevertheless, further research involving more samples of the same tissue should be performed to develop better techniques allowing preparing and sequencing the DNA with higher fidelity and reliability.

## Figures and Tables

**Table 1:** Somatic non-silent variants were detected in the examined patients in an ectopic (ECT) or eutopic (EU) endometrial tissue. Localization and type of mutation were described in concordance to Human Genome Variation Society (HGVS) nomenclature (variants are described on protein and cDNA level, “*”—stop codon). The allele frequency from all populations in the Genome Aggregation Database (gnomAD) was provided.

Sample	Gene	HGVS	gnomAD Allele Frequency
EEP001ECT	KCNH5	NM_139318.4:p.Gly808Val/c.2423G > T	0
EEP001ECT	MITF	NM_198159.2:p.Ser258Leu/c.773C > T	0.0000119
EEP001ECT EEP001EU	HERC2	NM_004667.5:p.Val3327Met/c.9979G > A	0.0345
EEP002ECT	KRAS	NM_033360.3:p.Gly12Asp/c.35G > A	0.00000401
EEP005ECT	ZNF804B	NM_181646.2:p.Glu1340Gln/c.4018G > C	0
EEP005ECT	CSMD3	NM_198123.1:p.Thr1246Met/c.3737C > T	0.0000639
EEP005ECT	CSMD3	NM_198123.1:c.8440 + 4C > T	0
EEP005ECT	NSD1	NM_022455.4:p.His918Tyr/c.2752C > T	0
EEP005ECT	NCOA1	NM_003743.4:p.Ala1081Val/c.3242C > T	0
EEP005ECT	MUC16	NM_024690.2:p.Gly8417Val/c.25250G > T	0
EEP005ECT	SMOX	NM_001270691.1:p.Ala356Thr/c.1066G > A	0
EEP005ECT	EWSR1	NM_013986.3:p.Gln146 */c.436C > T	0
EEP005ECT	NTRK2	NM_006180.4:p.Glu634Asp/c.1902G > C	0
EEP005ECT	PRDM9	NM_020227.2:p.Pro663Ser/c.1987C > T	0
EEP005EU	CASP8	NM_001080125.1:p.Glu71Gly/c.212A > G	0
EEP005EU	PSIP1	NM_001128217.1:p.Leu368Arg/c.1103T > G	0
EEP005EU	DNAH7	NM_018897.2:p.Gly91Val/c.272G > T	0
EEP005EU	FANCD2	NM_033084.3:p.Lys871Asn/c.2613A > C	0.000204
EEP005ECT EEP005EU	JAK2	NM_004972.3:p.Pro500_Pro501fs/c.1498_1499insC	0
EEP006ECT	ATRX	NM_000489.4:p.Gln1551 */c.4651C > T	0
EEP006ECT	KTN1	NM_001079521.1:p.Pro992_Pro993fs/c.2974_2975insC	0
EEP006ECT	CNOT1	NM_016284.4:p.Pro1254Ser/c.3760C > T	0.00000399
EEP006ECT	CHD2	NM_001271.3:c.-228T > C	0
EEP008ECT	BRIP1	NM_032043.2:p.Arg579His/c.1736G > A	0.0000278
EEP008EU	AR	NM_000044.3:p.Glu710Lys/c.2128G > A	0
EEP008EU	DMD	NM_004006.2:p.Gly3235Asp/c.9704G > A	0
EEP008EU	EPCAM	NM_002354.2:p.Gly79Trp/c.235G > T	0
EEP008EU	SMC3	NM_005445.3:p.Gly531Cys/c.1591G > T	0
EEP008EU	SYNE2	NM_182914.2:p.Leu6190Ile/c.18568C > A	0
EEP009ECT	TP53	NM_000546.5:p.Glu271Lys/c.811G > A	0
EEP009EU	COL1A1	NM_000088.3:p.Gly209Asp/c.626G > A	0
EEP009EU	DSCAM	NM_001389.3:p.Val1261Leu/c.3781G > C	0.0000201
EEP010ECT	PKD1L1	NM_138295.3:p.Gln122_Leu125del/c.363_374delACAGGCGCCTCT	0
EEP010ECT	ABL2	NM_007314.3:p.Ala114Glu/c.341C > A	0
EEP010EU	ETV5	NM_004454.2:p.Tyr429_Tyr430fs/c.1286_1287insA	0
EEP010EU	KDM6A	NM_021140.3:c.619 + 1_619 + 2delAAGT	0
EEP012ECT	RYR1	NM_000540.2:p.Thr1406Met/c.4217C > T	0.0000442
EEP012EU	AURKAIP1	NM_001127230.1:p.Ser72Arg/c.216C > A	0
EEP012EU	OBSCN	NM_001271223.2:p.Ala809Val/c.2426C > T	0.0000487
EEP012EU	HIP1	NM_005338.6:c.2466-4G > A	0
EEP014EU	FBN1	NM_000138.4:c.5788 + 7G > T	0
EEP014EU	SORCS1	NM_001013031.2:p.Val722Ala/c.2165T > C	0
EEP015ECT	ERBB3	NM_001982.3:p.Gly325Arg/c.973G > A	0
EEP015EU	LAMA2	NM_000426.3:p.Arg2604 */c.7810C > T	0.00000797
EEP015EU	MYO3A	NM_017433.4:p.Thr1501Lys/c.4502C > A	0
EEP015EU	RNASEL	NM_021133.3:p.Ala99Thr/c.295G > A	0
EEP015EU	RYR1	NM_000540.2:p.Gln240His/c.720G > T	0
EEP015EU	DNAH7	NM_018897.2:p.Ala1578Asp/c.4733C > A	0
EEP015EU	CTNNA1	NM_001903.3:c.1063-10834C > A	0
EEP015EU	PTCH1	NM_000264.3:p.Ser764Gly/c.2290A > G	0
EEP015EU	FAT3	NM_001008781.2:p.Ser2776Asn/c.8327G > A	0
EEP015EU	ATRX	NM_000489.4:p.Val194Ala/c.581T > C	0
EEP015EU	KLHL6	NM_130446.2:p.Arg308His/c.923G > A	0.000299
EEP015ECT EEP015EU	DYSF	NM_003494.3:p.Arg1041Cys/c.3121C > T	0.000168
EEP015ECT EEP015EU	TROAP	NM_005480.3:p.Ala235Asp/c.704C > A	0.0000398
EEP016ECT	MTUS2	NM_001033602.2:p.Arg468Gln/c.1403G > A	0.00075
EEP016EU	PIK3C2B	NM_002646.3:p.Val685Ile/c.2053G > A	0.0000837
EEP016EU	PTK2	NM_005607.4:p.Pro857Leu/c.2570C > T	0
EEP017EU	USP24	NM_015306.2:p.Ala909Val/c.2726C > T	0

## Data Availability

Data are available upon request via e-mail to the corresponding author.

## Supplementary Materials

The following are available online at https://www.mdpi.com/article/10.3390/cells10040749/s1, Supplementary Figure S1: WES results detected the same variants in both a eutopic (B) and ectopic (C) endometrial glands, but not in blood (A) in patients EEP001, EEP005, and EEP015. Supplementary Figure S2: ADS verification results: A—blood; B, C—eutopic endometrial glands; D, E—ectopic endometrial glands. Supplementary Figure S3: WES results detected variants only in an ectopic (C), but not in eutopic (B) endometrial glands and not in blood (A) in patients EEP002 and EEP009. Supplementary Table S1: List of genes included in our custom-gene panel.
