# Supplementary figures and images for: Epithelial Cells of Deep Infiltrating Endometriosis Harbor Mutations in Cancer Driver Genes
Source: Cells. 2021 Mar 29;10(4):749. doi: 10.3390/cells10040749 (PMC8065889; doi:10.3390/cells10040749)

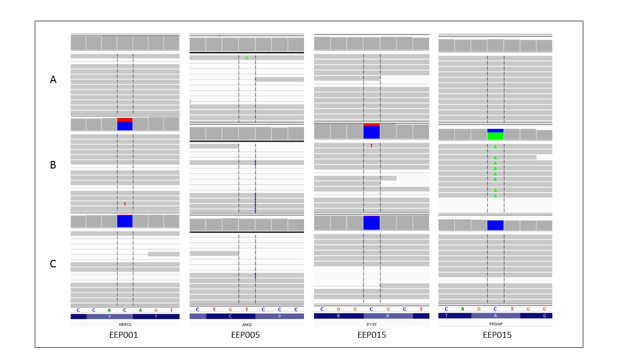

Supplement: Supplementary file 1 [file cells-10-00749-s001.zip › Suppl. Fig. S1.tiff]

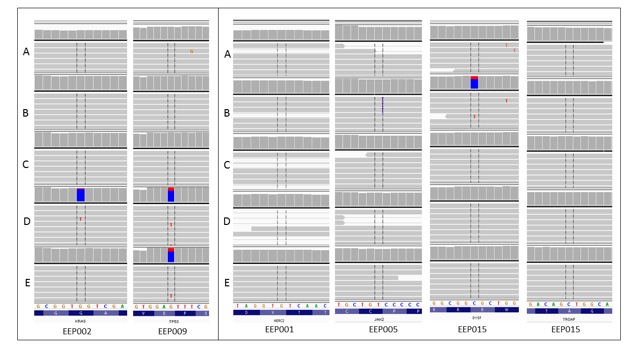

Supplement: Supplementary file 1 [file cells-10-00749-s001.zip › Suppl. Fig. S2.tiff]

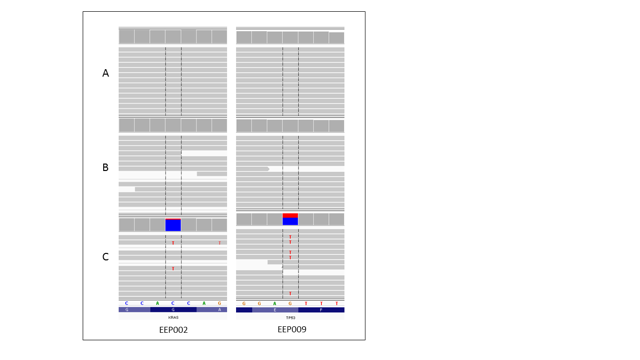

Supplement: Supplementary file 1 [file cells-10-00749-s001.zip › Suppl. Fig. S3.tiff]
